# Dynamic molecular and cellular characteristics of VSX2-positive retinal progenitor cells in human retinal organoids

**DOI:** 10.1186/s13287-025-04700-z

**Published:** 2025-10-28

**Authors:** Dandan Zheng, Yuan Wang, Yuanyuan Guan, Ping Xu, Bingbing Xie, Guanjie Gao, Xiaoming Yu, Fuhua Peng, Mengqing Xiang, Xiufeng Zhong

**Affiliations:** 1 State Key Laboratory of Ophthalmology, Zhongshan Ophthalmic Center, Sun Yat-sen University, Guangdong Provincial Key Laboratory of Ophthalmology and Visual Science, Guangzhou, 510060 China; 2 Department of Ophthalmology, Nanfang Hospital, Southern Medical University, Guangzhou, China; 3 The Key Laboratory for Stem Cells and Tissue Engineering, Ministry of Education, Zhongshan School of Medicine, Sun Yat-sen University, Guangzhou, China; 4 Guangzhou Genedenovo Biotechnology Co., Ltd, Guangzhou, 511400 China; 5 Department of Neurology, The Third Affiliated Hospital, Sun Yat- sen University, Guangzhou, 510000 China

**Keywords:** Retinal progenitor cell, VSX2, Retinal organoid, Cluster of differentiation biomarker, Retinal degenerative disease, Cell therapy

## Abstract

**Background:**

The lack of understanding of the molecular and cellular characteristics of human retinal progenitor cells (RPCs) has hindered their application in cell therapy for retinal degenerative diseases. This study aims to employ retinal organoids (ROs) derived from a VSX2-enhanced green fluorescent protein (eGFP) reporter human induced pluripotent stem cell (hiPSC) line for positive selection of human RPCs, investigate their features, and facilitate their applications.

**Methods:**

hiPSCs were differentiated into three-dimensional ROs following established protocols. The fidelity of the VSX2-eGFP reporter was confirmed through immunostaining. Fluorescence-activated cell sorting was employed to select VSX2-eGFP-positive (+) cells at distinct developmental stages, followed by bulk RNA sequencing (RNA-seq) analysis to assess their transcriptome profile. Immunostaining and flow cytometry were utilized to validate the identity of VSX2-eGFP+ cells and potential cluster of differentiation (CD) biomarkers for identifying human RPCs.

**Results:**

hiPSCs were successfully differentiated into ROs containing abundant RPCs. The spatiotemporal activity of the VSX2-eGFP reporter recapitulated the dynamic expression of endogenous VSX2 protein. Compared to VSX2-eGFP-negative (-) cells, VSX2-eGFP+ cells mainly exhibited characteristics of RPCs at early stages of retinal development and of bipolar cells at late stages. RNA-seq analysis revealed transcriptional heterogeneity within VSX2-eGFP+ cells across four distinct developmental stages. Moreover, the dynamic expression of 394 known CD biomarkers in VSX2-eGFP+ cells at distinct developmental stages was analyzed herein for the first time. One CD biomarker, TNFRSF1B, which has never been reported to be expressed in RPCs, was found to be highly expressed in RPCs at the early stages and might serve as a candidate CD biomarker for sorting RPCs.

**Conclusions:**

This study provides valuable insights into the molecular and cellular characteristics of human RPCs, especially their expression profiles of CD biomarkers, laying a foundation for research on retinal development and the clinical translation of hiPSC-derived RPCs.

**Supplementary Information:**

The online version contains supplementary material available at 10.1186/s13287-025-04700-z.

## Background

Retinal degenerative diseases (RDs) encompass a group of retinal disorders that cause blindness and are characterized by the loss of photoreceptor cells, such as age-related macular degeneration and retinitis pigmentosa, affecting millions of individuals worldwide [1, 2]. Currently, there are no effective therapeutic interventions for RDs. Cell therapy aimed at replacing damaged photoreceptor cells, thereby restoring visual function, holds great promise in addressing this unmet need [3, 4].

Human retinal progenitor cells (RPCs) are recognized as one of the most promising types of donor cells for RD cell therapy because of their capacity for self-renewal and differentiation into all major types of retinal cells [5, 6]. However, their application has been impeded by the limited availability and ethical controversies associated with RPCs isola*t*ed from the human fetal retina. Recent advancements in human pluripotent stem cells (hPSCs), including human embryonic stem cells (hESCs) and human induced pluripotent stem cells (hiPSCs), and three-dimensional retinal organoid (RO) technologies now offer a reliable source for obtaining human RPCs [7–9]. Initial studies have shown that RPCs isolated from hPSC-derived ROs can survive and differentiate into various retinal cell types, including photoreceptor cells, following subretinal transplantation, indicating the therapeutic potential of these cells [10].

However, the challenge persists in selectively enriching RPCs while preventing contamination with other cell types. Compared with genetically tagged donor cells, cells sorted on the basis of cell surface markers are deemed more appropriate for clinical applications due to their lower potential toxicity [11–14]. This approach enables the isolation of subsets of cells from complex cultures without damaging target cells. However, many surface markers lack commercially available antibodies for clinical use. Cluster of differentiation (CD) biomarkers, a category of proteins or glycoproteins on the cell membrane, are economical and convenient for direct application in preclinical and clinical studies, serving as favorable markers for cell identification and separation [15–18]. Nevertheless, although a few CD markers have been reported to label RPCs in animal studies, it remains unclear whether these findings can be replicated in human cells [19–21]. Furthermore, recent studies have revealed heterogeneity among RPCs at specific and distinct stages [22–24], highlighting the importance of selecting RPCs at favorable stages for transplantation. However, the dynamic expression profiles of cell surface markers in human RPCs remain inadequately characterized.

Visual system homeobox 2 (VSX2), also known as CHX10, is widely recognized as a canonical marker for the identification of RPCs [25–27]. In a previous study, we successfully established a hiPSC line harboring a VSX2-enhanced green fluorescent protein (VSX2-eGFP) reporter [28]. Here, we employed this hiPSC line in combination with transcriptome analysis to characterize the cellular and molecular properties of human RPCs at different stages. First, we observed the spatial and temporal activity of VSX2-eGFP in the RO system during neuroretinal determination, differentiation, and maturation from differentiation day (D) 0 to D225. Second, we explored the dynamic changes in the cellular and molecular characteristics of human VSX2-eGFP-positive (+) retinal cells. Finally, we analyzed the dynamic expression profiles of 394 CD biomarkers at distinct stages and extracted potential biomarkers for RPC sorting. We anticipate that this study will contribute to advancements in cell therapy for RDs.

## Methods

### Generation of ROs from hiPSCs

This study utilized four hiPSC lines: BC1, VSX2-eGFP, BRN3B-GFP and UC3X18. The BC1-hiPSC line was derived from anonymous bone marrow CD34+ cells, which was approved by the Institutional Review Board (IRB) of the Johns Hopkins University [29]. The VSX2-eGFP hiPSC line was engineered from BC1-hiPSCs [28]. The BRN3B-GFP hiPSC line was generated from the UiPSC-001 hiPSC line, which was derived from anonymous urine cells and approved by the IRB of Sun Yat-sen University [30]. The UC3X18 hiPSC line was generated by our lab using anonymous urine cells as previously described [31], which was approved by the IRB of Sun Yat-sen University (2017KYPJ061-7) with informed consent. hiPSCs were maintained on Matrigel (Corning)-coated plates with mTeSR1 medium (Stem Cell Technologies) and passaged every 4–6 days at 80% confluence. The differentiation of hiPSCs into ROs was performed according to our published protocols [32].

### Immunostaining

hiPSC-derived cultures at very-early stages (D16) on coverslips, VSX2-eGFP+ cells sorted from early-stage (D31) ROs and seeded on coverslips for one day, and ROs at distinct stages were all fixed in 4% PFA. Tissue cryopreservation, sectioning and immunostaining were performed as previously described [33]. All the antibodies used are listed in Table S1. 4’,6-Diamidino-2-phenylindole (DAPI; Dojindo Molecular Technologies) was used for nuclear counterstaining. An LSM 880 confocal microscope, an Axio Scan 21 microscope and a HAL 100 microscope (Zeiss) were used for the acquisition of bright field and fluorescence images. Images were processed in Zen2.3 (Zeiss) and Photoshop CS5 (Adobe).

### Real-time quantitative polymerase chain reaction (RT-qPCR)

Total RNA extraction was performed with TRIzol Reagent (Invitrogen). A PrimeScript RT Reagent Kit with gDNA Eraser (Perfect Real Time) (Takara, Shiga Prefecture, Japan) was used to remove potential DNA contamination. The RNA quality was evaluated via a SMA4000 Ultra Micro Spectrophotometer (Merinton). Reverse transcription was performed via the PrimeScript RT Reagent Kit with gDNA Eraser (Perfect Real Time) (Takara). qPCR was performed with Hieff UNICON qPCR SYBR Green Master Mix (YeaSen) on an ABI 7300 fluorescence quantitative PCR instrument (Thermo Fisher Scientific). Predenaturation was performed at 95 °C for 3 min. Cycles (40 cycles) were run at 95 °C denaturation for 10 s, at 60 °C annealing for 34 s and at 72 °C extension for 34 s. The primers used are listed in Table S2. The 2^−ΔΔCt^ method was used to analyze the data. GAPDH served as an internal control, and BC1-hiPSCs served as a reference.

### Fluorescence-activated cell sorting (FACS)

All cultures in 100-mm culture dishes at D28 or neural retinas (NRs) isolated from ROs at D55, 118 and 170 were collected and dissociated into single cells via a papain dissociation system (Worthington Biochemical) according to the manufacturer’s instructions. The cell suspension was centrifuged at 400 × g for 10 min (Allegrax-15R, Beckman Coulter), and the cell pellets were resuspended in sorting buffer (PBS containing 1 mM EDTA (GIBCO) and 2% [vol/vol] FBS). FACS was performed as previously described [14]. The cells were sorted at 4 °C with a BD FACSAria fusion cell sorter (Becton Dickinson). eGFP+ and eGFP- cells were separately collected in collection buffer (medium plus 50% [vol/vol] FBS). FSC-A/SSC-A, FSC-A/FSC-H were examined in all the cells to obtain live single cells.

### RNA sequencing (RNA-seq) and data analysis

RNA-seq was performed as previously described [14, 33]. D60 ROs and eGFP+ and eGFP- cells pooled at D28, D55, D118 and D170 were collected in TRIzol reagent (Invitrogen) and stored at -80 °C until they were sent to Gene Denovo Biotechnology Co. (Guangzhou, China) for RNA extraction, library preparation, sequencing and data analyses. Total RNA was extracted via a TRIzol reagent kit (Invitrogen). RNA quality was assessed on an Agilent 2100 Bioanalyzer (Agilent Technologies). Oligo (dT) beads were used to isolate the poly mRNA from the total RNA. The enriched mRNA was fragmented and reverse transcribed into cDNA via random primers. After synthesis of the second strand, the cDNA was purified, end-repaired and ligated to Illumina sequencing adapters. The ligation products were size selected via agarose gel electrophoresis, amplified via PCR, and sequenced via the Illumina NovaSeq 6000 platform. The raw data were processed via the fastp tool (version 0.18.0) and short-read alignment tool Bowtie2 (version 2.2.8) to obtain clean reads. Paired-end clean reads were mapped to the reference genome via HISAT2.2.4. The mapped reads of each sample were assembled via StringTie (version 1.3.1). For each transcriptional region, the fragment per kilobase of transcript per million mapped reads (FPKM) value was calculated via RSEM to quantify the expression levels of each gene. Differential expression analysis was performed via DESeq2 or edgeR. Genes or transcripts with a false discovery rate (FDR) < 0.05 and absolute fold change ≥ 2 were considered differentially expressed genes (DEGs). Gene set enrichment analysis (GSEA) was performed via GSEA and MSigDB software. The gene sets with an absolute normalized enrichment score (NES) value ≥ 1.0, nominal (NOM) p value ≤ 0.05, and FDR q value ≤ 0.25 were considered significant. The accession number for the RNA-seq data reported in this paper is GEO: GSE185475.

### Flow cytometry

NRs isolated from ROs at D55 were dissociated into single cells using the papain dissociation system (Worthington Biochemical). Cells were fixed in 1% PFA for 15 min at room temperature (RT), washed, blocked, and permeabilized for 30 min at RT. Subsequently, cells were incubated with primary antibodies (1 µg per 10⁶ cells) against Ki67 (Abcam, ab15580), VSX2 (Millipore, ab9016), and TNFRSF1B (Thermo Scientific, MA5-32618) for 1 h at RT. Following PBS washes, cells were incubated with Alexa Fluor-conjugated secondary antibody (1:500, Thermo Fisher Scientific) for 30 min at RT. Washed cells were analyzed using an LSRFortessa flow cytometer (BD Biosciences). Single cells were gated using FSC-A/SSC-A parameters to exclude debris, with doublet discrimination performed using FSC-A versus FSC-H measurements. Cells without primary antibody incubation were used as a negative control. Data analysis was performed using FlowJo_v10.8.1 software.

### Statistical analyses

Unless otherwise stated, the data are presented as the mean ± SD. When only two independent groups were compared, significance was determined by a two-tailed unpaired t test. A p value < 0.05 was considered significant. The analysis was performed in GraphPad Prism v.9.2.0.

## Results

### The VSX2-eGFP reporter recapitulated the dynamic expression of endogenous VSX2

**Fig. 1:**
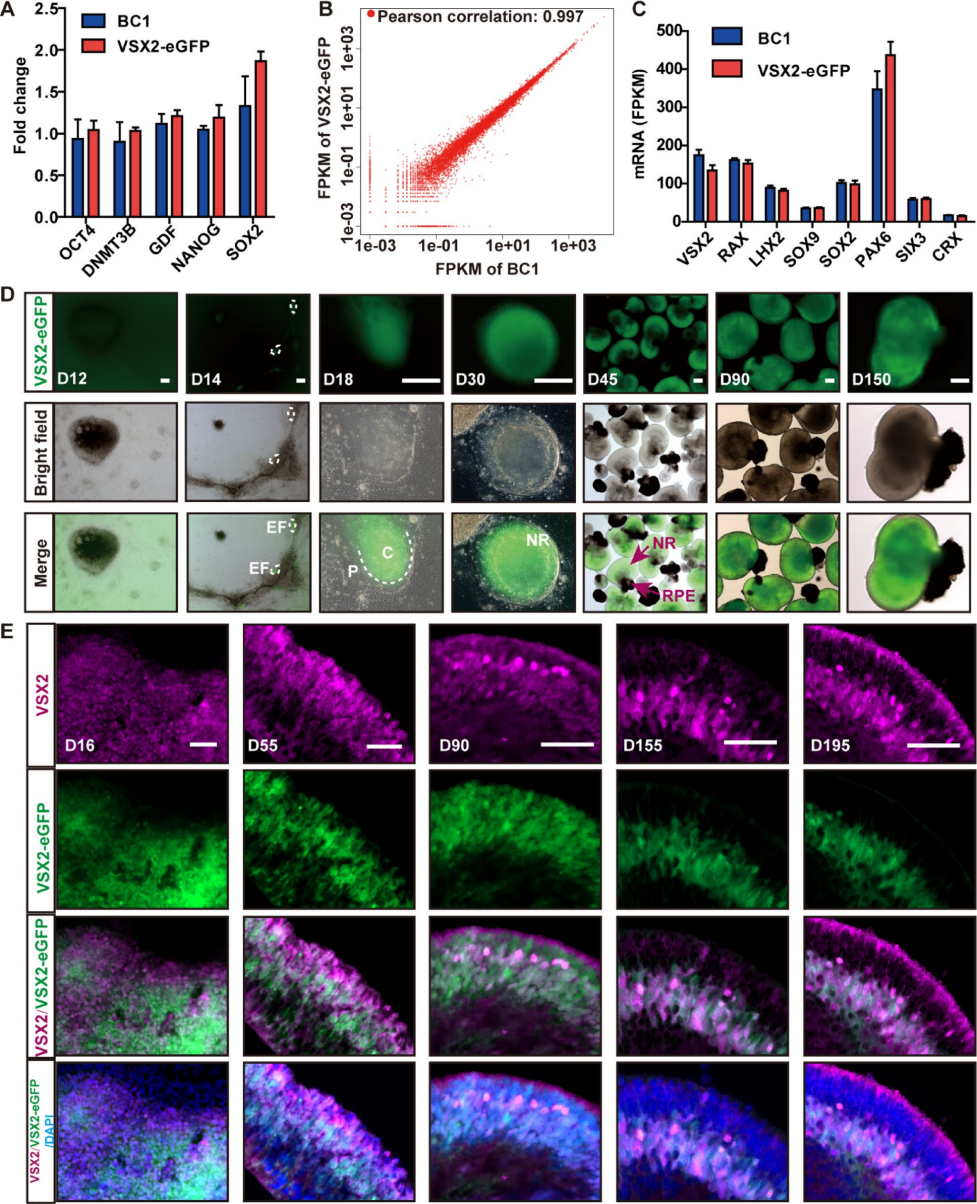
Spatiotemporal expression of the VSX2-eGFP reporter in the RO system. **A** Bar chart showing RT-qPCR analysis of the reporter and parental hiPSCs. **B** Scatter plot showing the Pearson’s correlation between ROs derived from both reporter hiPSCs and parental hiPSCs at D60. **C** Bar chart showing the expression levels of retinal lineage genes in ROs derived from both reporter and parental hiPSCs at D60.** D** Fluorescent and bright-field images showing the spatiotemporal expression patterns of the VSX2-eGFP reporter in the RO system from D0 to D150. **E** Immunostaining with an anti-VSX2 antibody showed co-localization of the VSX2-eGFP reporter and endogenous VSX2 at various designated time points. C: center; P: periphery. Scale bars: 200 μm (**D**), 50 μm (**E**)

A VSX2-eGFP hiPSC line was established in our previous study by introducing the eGFP sequence at the reading frame terminator of exon 5 of *VSX2* [28]. The VSX2-eGFP hiPSCs robustly maintained pluripotency similar to that of the parental hiPSCs and successfully differentiated into ROs (Figs. 1A and S1A). RNA-seq analysis of D60 ROs revealed a high degree of correlation between the reporter and parental hiPSC-derived ROs, with a Pearson correlation coefficient of 0.997 (Fig. 1B). Furthermore, there was no significant difference in the expression levels of retinal lineage genes such as *VSX2*, *RAX*, *LHX2*, *SOX9*, *SOX2*, *PAX6*, *SIX3*, and *CRX* between the reporter and parental hiPSC-derived ROs, excluding the detrimental effects of the genetic engineering procedure (Fig. 1C).

With light and inverted fluorescence microscopes, the spatiotemporal activity of VSX2-eGFP was longitudinally observed in the RO system during retinal development from D0 to D225 (Figs. 1D, S1B–D). From D12 to D14, VSX2-eGFP was initially detected in eye field-like structures (EFs) (Figs. 1D and S1B). From D14 to D30, the EFs gradually developed into optic cup-like structures (OCs) consisting of a NR-like domain and a retinal pigment epithelium (RPE)-like domain (Fig. 1D). VSX2-eGFP exhibited a distinct distribution within the central NR domain of OCs rather than at the periphery (Fig. 1D). After D30, manual lifting of OCs resulted in the formation of ROs comprising an NR ring attached with more or less pigmented RPE cells (Fig. 1D). In these ROs, exclusive expression of VSX2-eGFP was observed solely in the NR layer at all tested stages but not in RPE cells (Fig. 1D). From D90 to D225, there was a gradual decrease in the fluorescence density of VSX2-eGFP, specifically in the outermost layer of the NR (Fig. 1D, S1C–D). Immunostaining demonstrated that the dynamic changes in VSX2-eGFP reporter signals largely mirrored the alterations in endogenous VSX2 expression, as revealed by anti-VSX2 antibody staining in NRs from D16 to D195 (Fig. 1E). Although a strong correlation was established, a trace of transient VSX2-eGFP signals was also observed in the innermost layer of NRs in D55 ROs, where nascent retinal cells had just differentiated from RPCs; these signals progressively diminished with maturation and became minimal in later stages (Fig. 1E).

Overall, the activity of the VSX2-eGFP reporter reflected the endogenous expression pattern of VSX2 and thus validated its utility as a fluorescent marker for identifying and sorting VSX2+ cells.

### VSX2-eGFP+ cells primarily displayed characteristics of RPCs at early stages and of bipolar cells at late stages

To systematically characterize VSX2-eGFP+ cells across distinct developmental stages defined according to previous studies [34] with some modifications, we first compared their transcriptional profiles with VSX2-eGFP- cells.

**Fig. 2:**
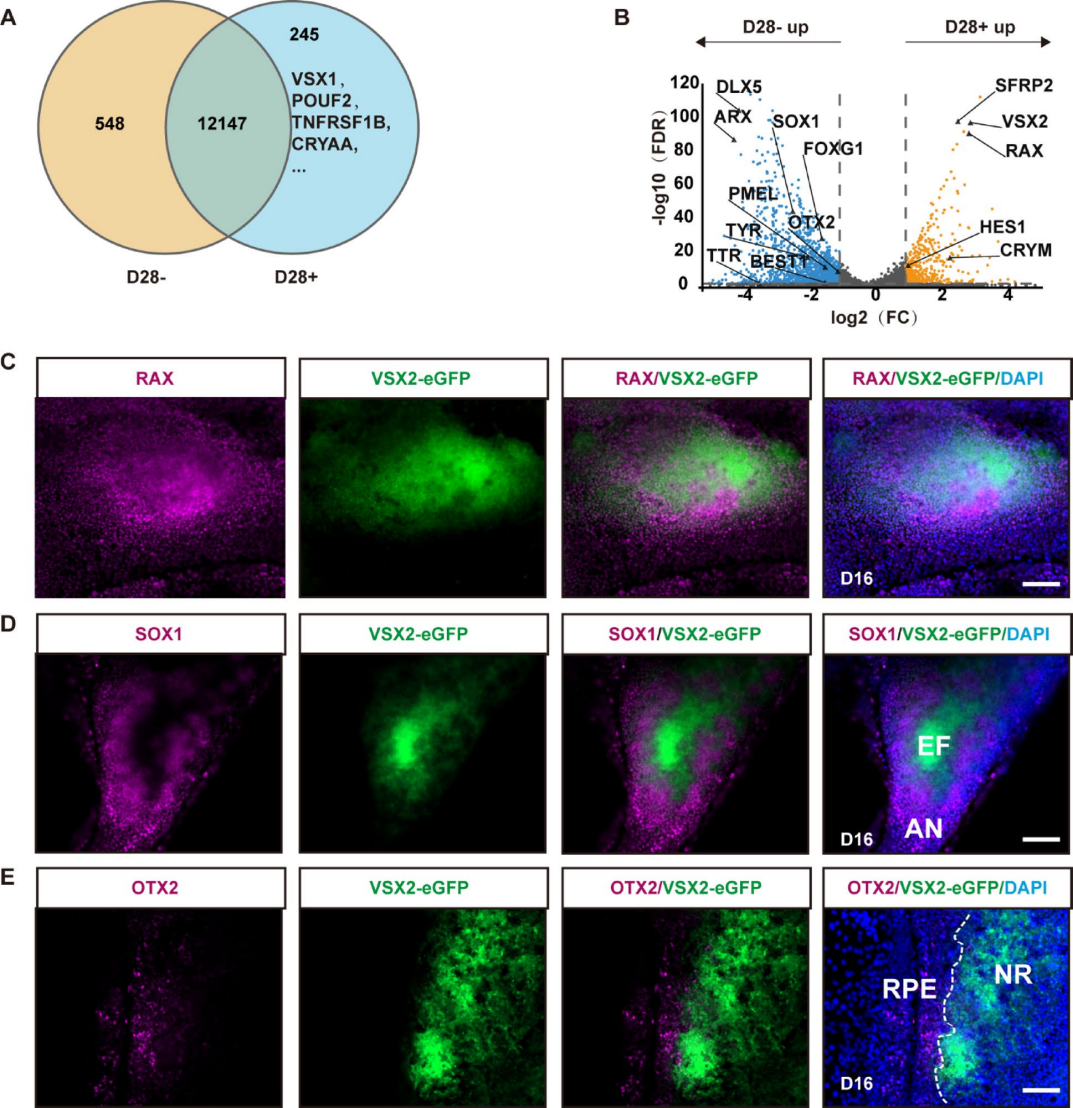
Cellular characteristics of VSX2-eGFP+ cells at very-early stages. **A** Venn diagrams showing the numbers of SEGs (FPKM ≥ 1) in VSX2-eGFP+ cells sorted at D28. **B** Volcano plots showing DEGs between VSX2-eGFP+ and VSX2-eGFP- cells sorted at D28. Up: upregulated; Down: downregulated; FC: fold change. **C**–**E** Representative immunostaining images showed that most of the VSX2-eGFP+ cells in D16 cultures expressed RPC marker RAX (**C**), but exhibited no or faint expression of telencephalic-specific marker SOX1 (**D**) or RPE marker OTX2 (**E**). AN: anterior neuroepithelial. Scale bars: 100 μm (**C**–**E**)

At very-early stages (D14–D30), which is characterized by the formation of OCs consisting of nascent neural retina and RPE, 245 specifically expressed genes (SEGs, FPKM ≥ 1) and 1,735 differentially expressed genes (DEGs) were identified in VSX2-eGFP+ cells sorted from D28 cultures, as compared with VSX2-eGFP- cells (Fig. 2A–B). Early RPC-related genes such as *VSX2*, *RAX*, *SFRP2*, *CYRM*, and *HES1* were upregulated in VSX2-eGFP+ cells, whereas genes associated with brain development (*FOXG1*, *SOX1*, and *ARX*) and RPE cells (*OTX2* and *BEST1*) were downregulated (Fig. 2B). Immunostaining revealed that the majority of VSX2-eGFP+ cells in D16 cultures were positive for the RPC markers VSX2 and RAX (Figs. 1E and 2C), but exhibited negative or faint expression of the telencephalic-specific marker SOX1 (Fig. 2D) and the RPE marker OTX2 (Fig. 2E). Collectively, these results revealed that VSX2-eGFP + cells primarily exhibited characteristics of RPCs at the very-early stage.

**Fig. 3:**
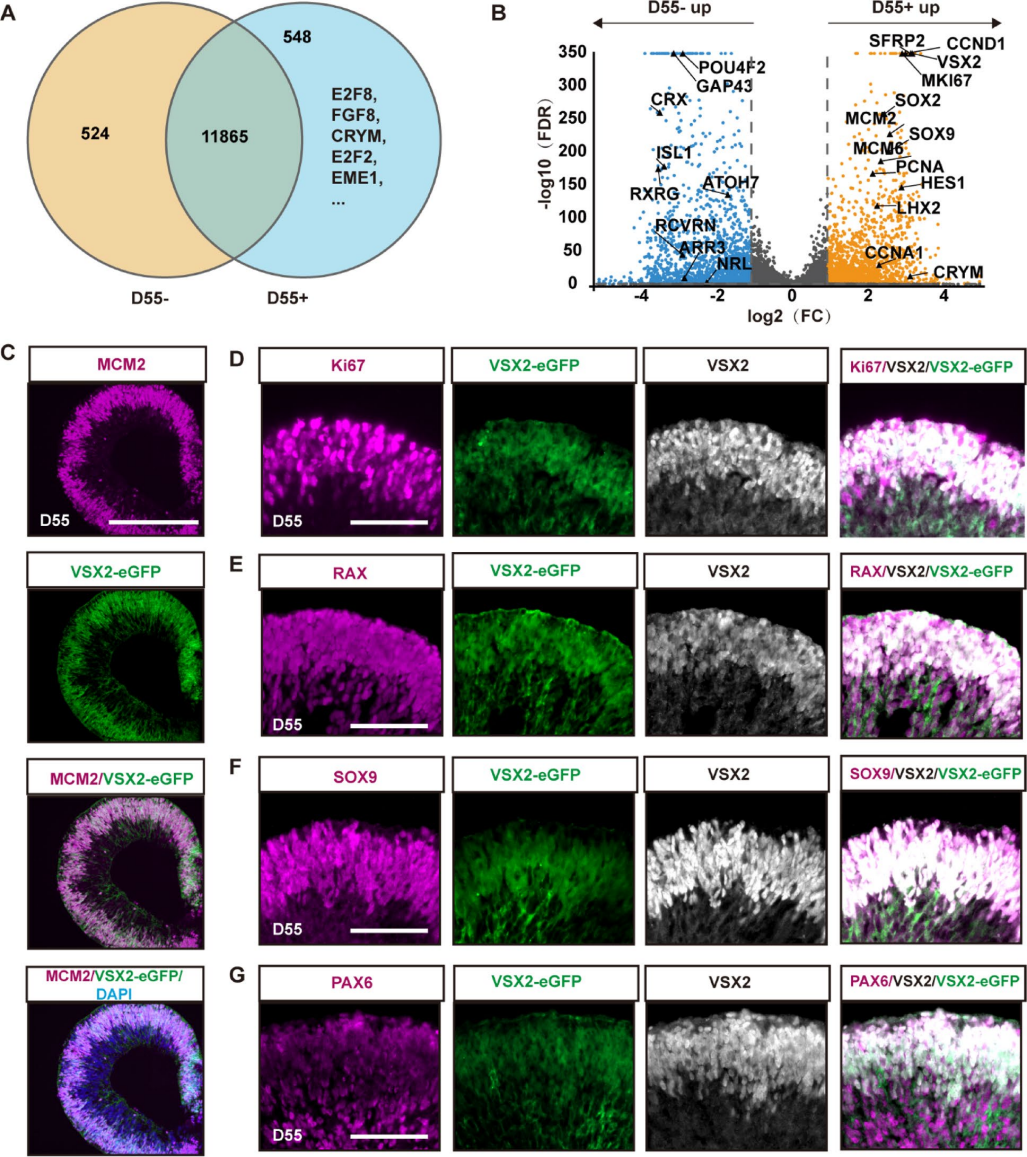
Cellular characteristics of VSX2-eGFP+ cells at early stages. **A** Venn diagrams showing the numbers of SEGs in VSX2-eGFP+ cells sorted at D55. **B** Volcano plots showing DEGs between VSX2-eGFP+ and VSX2-eGFP- cells sorted at D55. **C**–**G** Representative immunostaining images showed most of the VSX2-eGFP+ cells in D55 ROs expressed both the proliferation markers MCM2 (**C**) and Ki67 (**D**), and the RPC markers RAX (**E**), SOX9 (**F**), and PAX6 (**G**). Scale bars: 50 μm (**C**), 100 μm (**D**–**G**)

At early stages (D31–D90), manually lifted OCs develop into ROs, which consist of an NR ring and attached RPE cells, followed by the emergence of early-born retinal cells (retinal ganglion cells, cones, horizontal/amacrine cells) in the NRs. There were 548 SEGs in the VSX2-eGFP+ cells sorted from D55 ROs (Fig. 3A). Differential expression analysis revealed 3,550 DEGs between VSX2-eGFP+ and VSX2-eGFP- cells (Fig. 3B). Compared with VSX2-eGFP- cells, VSX2-eGFP+ cells highly expressed RPC-related genes such as *VSX2*, *SFRP2*, *CRYM*, *SOX9*, *SOX2*, *LHX2*, and *HES1* as well as cell proliferation-associated genes such as *MKI67*, *PCNA*, *MCM2*, *MCM6*, *CCNA1*, and *CCND1*. However, VSX2-eGFP+ cells exhibited lower expression of both retinal ganglion cell-related genes (*ATOH7* and *POU4F2*), and photoreceptor cell marker genes (*CRX*, *RXRG*, and *NRL*) (Fig. 3B). Immunostaining showed that most of the VSX2-eGFP+ cells in D55 ROs expressed cell proliferation markers MCM2 and Ki67 (Fig. 3C–D), along with the RPC markers RAX, SOX9, and PAX6 (Fig. 3E–G), indicating that these cells were primarily proliferating RPCs.

**Fig. 4:**
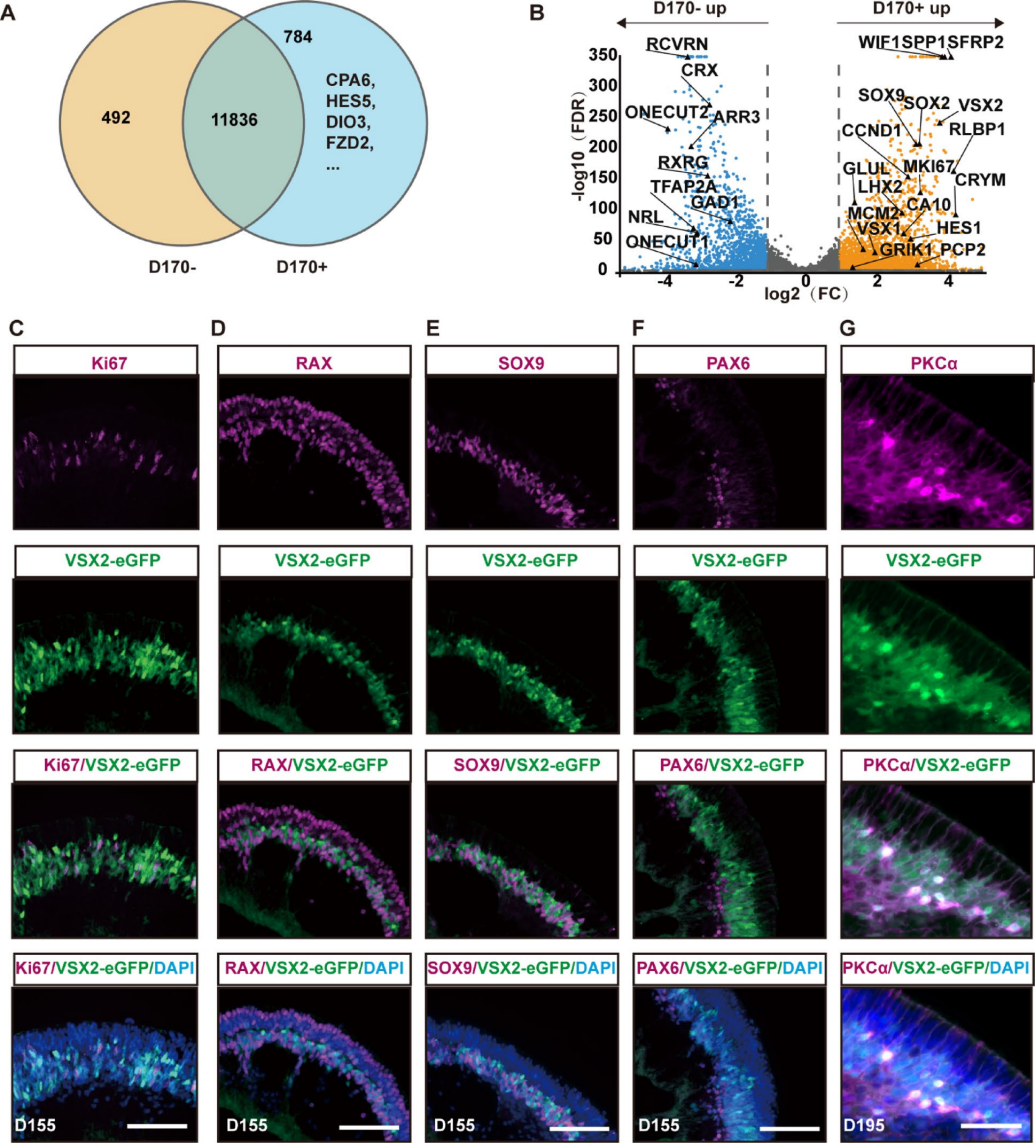
Cellular characteristics of VSX2-eGFP+ cells at late stages. **A** Venn diagrams showing the numbers of SEGs in VSX2-eGF+ cells sorted at D170. **B** Volcano plots showing DEGs between VSX2-eGFP+ and VSX2-eGFP- cells sorted at D170. **C**–**G** Representative immunostaining images showed that the majority of VSX2-eGFP cells at D155 and D195 did not express RPC-related markers Ki67 (**C**), RAX (**D**), SOX9 (**E**), and PAX6 (**F**), but expressed bipolar cell markers PKCα (**G**). Scale bars: 100 μm (**C**–**F**), 50 μm (**G**)

At late stages (after D150), all major retinal cell types including late-born cells (rods, bipolar cells, and Müller glial cells) emerge. VSX2-eGFP+ cells were found primarily in the intermediate layer of the NRs. There were 784 SEGs in the VSX2-eGFP+ cells sorted from D170 ROs (Fig. 4A). Differential expression analysis of VSX2-eGFP+ and VSX2-eGFP- cells revealed 4,182 DEGs (Fig. 4B). Compared with VSX2-eGFP- cells, VSX2-eGFP+ cells upregulated RPC related genes (*VSX2*, *SOX2*, *SOX9*, *LHX2*, *SFRP2*, *CRYM*, *HES1*, *MKI67*, *PCNA*, and *MCM2*), bipolar cell-related genes (*PCP2*, *VSX1*, *CA10*, and *GRIK1*), and Müller glial cell-related genes (*GLUL* and *RLBP1*) (Fig. 4B), but downregulated amacrine and horizontal cell-related genes (*TFAP2A*, *ONECUT1*, and *ONECUT2*) and photoreceptor cell marker genes (*CRX*, *RXRG*, and *NRL*) (Fig. 4B). Immunostaining revealed that the majority of VSX2-eGFP+ cells located in the inner nuclear layer of NRs at late stages (D155 and D195), did not express RPC-related markers Ki67, RAX, SOX9, or PAX6 (Fig. 4C–F), but expressed bipolar cell markers PKCα and VSX2 (Figs. 1E and 4G). Additionally, all VSX2-eGFP+ cells did not express the pan-photoreceptor marker RCVRN, the rod marker rhodopsin or the cone marker arrestin-C (Fig. S2A–C). VSX2-eGFP reporter was also negative in amacrine cells (AP2α+) and horizontal cells (PROX1+ PAX6+) (Fig. S2D–E). All the above findings suggested that most of the VSX2-eGFP+ cells at this stage exited the cell cycle and primarily exhibited characteristics of bipolar cells.

Collectively, these data indicate that VSX2-eGFP+ cells exhibit characteristics of RPCs at early stages of retinal development and of bipolar cells at late stages, which is consistent with the findings of previous studies [35, 36].

### VSX2-eGFP+ cells exhibited transcriptional heterogeneity across distinct developmental stages

**Fig. 5:**
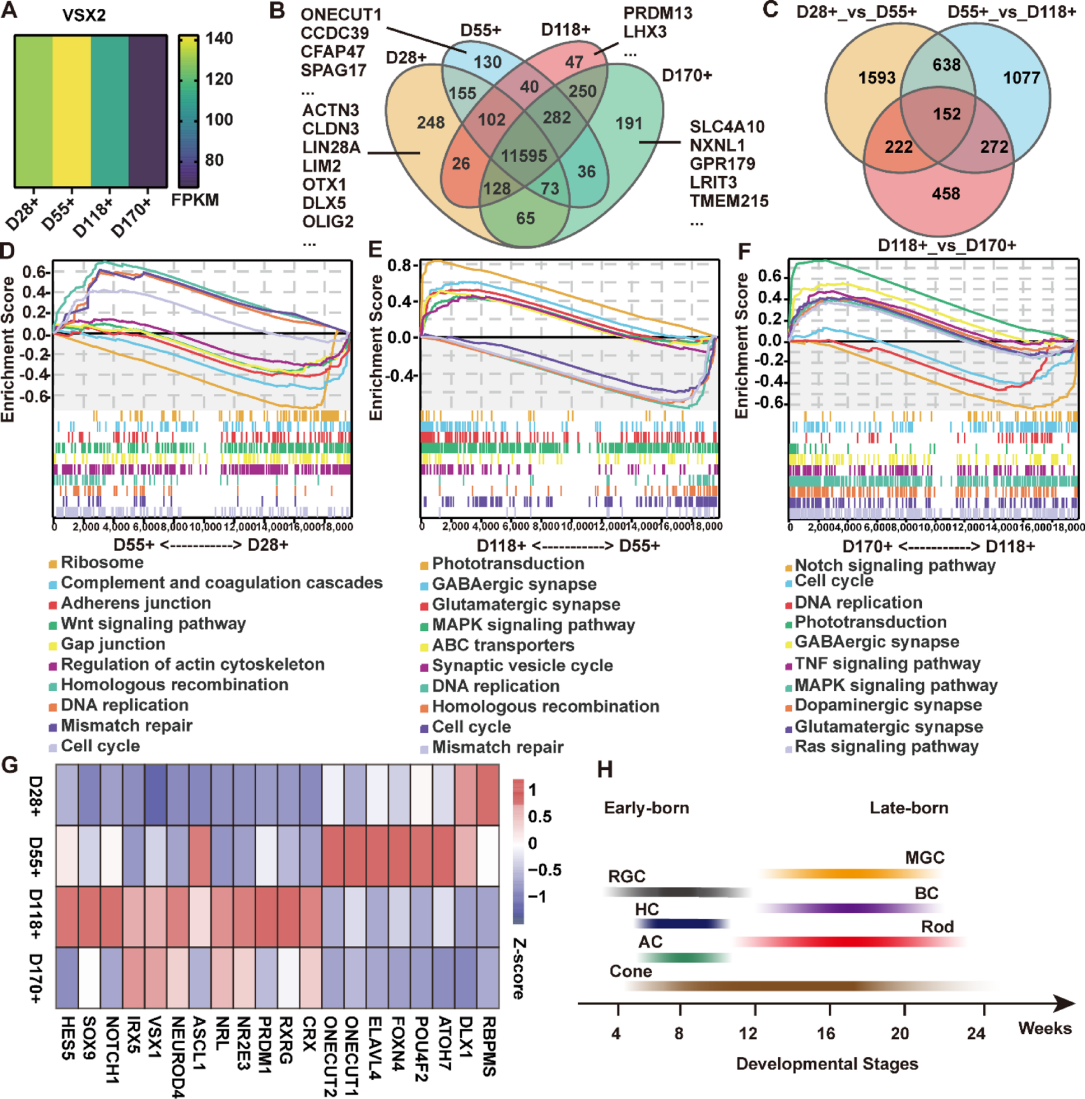
Transcriptome characteristics of VSX2-eGFP+ cells at distinct stages. **A** Heatmap showing the expression level of VSX2 in VSX2-eGFP+ cells at distinct stages. **B** Venn diagram showing the numbers of SEGs in VSX2-eGFP+ cells at distinct stages. **C** Venn diagram showing the numbers of DEGs between VSX2-eGFP+ cells at any two consecutive stages. **D**–**F** GSEA revealing the enriched gene sets in the Kyoto Encyclopedia of Genes and Genomes collection of VSX2-eGFP+ cells at distinct stages. Each line represents one gene set with a unique color. **G** Heatmap showing the expression dynamics of known transcription factors and genes involved in the competence, specification and differentiation of different retinal cell types. **H** Schematic diagram showing the birth order of different retinal cells

Next, we compared the transcriptomic differences in VSX2-eGFP+ cells across distinct developmental stages. First, we found that VSX2 expression levels were comparable in VSX2-eGFP+ cells sorted at D28, D55, and D118. However, expression was reduced in VSX2-eGFP+ cells sorted at D170 compared with these three earlier time points (Fig. 5A). Additionally, stage-specific genes were identified in VSX2-eGFP+ cells at all examined time points. At D28, 248 SEGs (*LIN28A*, *LIM2*, and *OLIG2*) were detected in VSX2-eGFP+ cells; at D55, 130 SEGs (*ONECUT1*, *CCDC39*, and *CFAP47*); at D118, 47 SEGs (*PRDM13* and *LHX3*); and at D170, 191 SEGs (*SLC4A10*, *NXNL1*, and *GPR179*) (Fig. 5B).

Differential expression analysis revealed 2,605 DEGs between VSX2-eGFP+ cells at D28 and D55 (Fig. 5C). GSEA revealed that pathways such as the cell cycle and DNA replication were enriched in VSX2-eGFP+ cells at D55, whereas pathways such as ribosome and adherens junction were enriched in VSX2-eGFP+ cells at D28 (Fig. 5D). A total of 2,139 DEGs were present between VSX2-eGFP+ cells at D55 and D118 (Fig. 5C). Pathways related to phototransduction, glutamatergic synapse, and the synaptic vesicle cycle were enriched in the VSX2-eGFP+ cells at D118, whereas pathways related to the cell cycle and DNA replication were enriched at D55 (Fig. 5E). A total of 1,104 DEGs were found between VSX2-eGFP+ cells at D118 and D170 (Fig. 5C). Pathways such as the glutamatergic synapse and phototransduction were enriched at D170 (Fig. 5F), whereas pathways such as the Notch signaling pathway, cell cycle, and DNA replication were enriched at D118 (Fig. 5F).

The dynamic expression patterns of known genes involved in the competence, specification, and differentiation of different retinal cell types revealed the different neurogenic potentials or cell fates of VSX2-eGFP+ cells at distinct developmental stages. VSX2-eGFP+ cells at D28 and D55 highly expressed early-born retinal cell-related genes, including retinal ganglion cell marker genes (*DLX1*, *ATOH7*, and *POU4F2*) and amacrine/horizontal cell marker genes (*FOXN4*, *ELAVL4*, *ONECUT1,* and *ONECUT2*) (Fig. 5G). Genes related to cones, such as *CRX* and *RXRG*, were upregulated from D55 onward and enriched at D118. After D55, VSX2-eGFP+ cells highly expressed late-born retinal cell genes, including rod markers (*PRDM1*, *NR2E3*, and *NRL*), bipolar cell markers (*ASCL1*, *NEUROD4*, *VSX1*, and *IRX5*), and Müller glial cell markers (*NOTCH1*, *SOX9*, and *HES5*) (Fig. 5G).

Overall, VSX2-eGFP+ cells exhibited significant transcriptional heterogeneity across distinct developmental stages, which was consistent with the results of previous studies [22–24].

### Dynamic expression of 394 CD biomarkers in VSX2-eGFP+ cells revealed potential markers for human RPC sorting

**Fig. 6:**
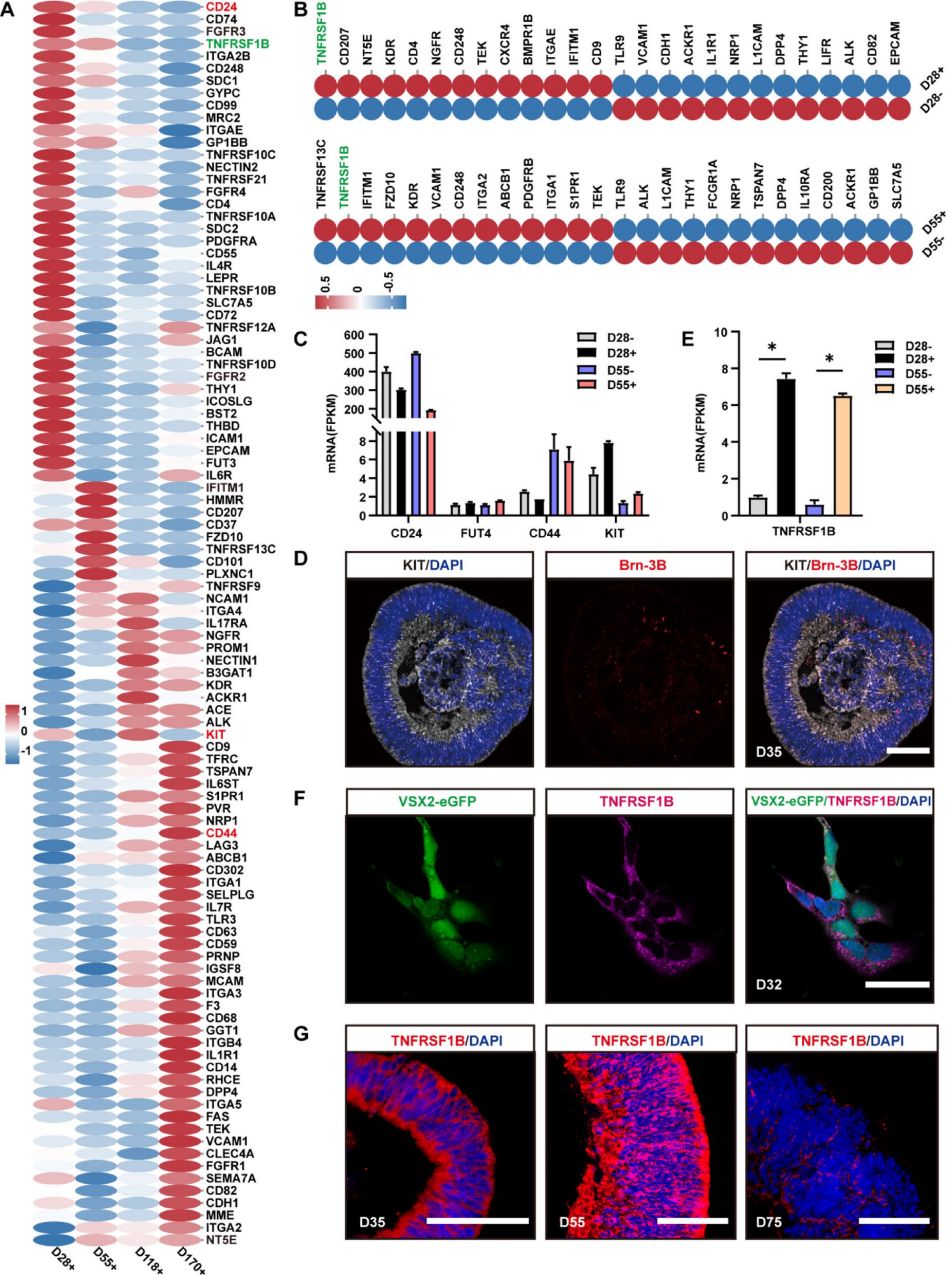
Expression patterns of CD biomarkers in VSX2-eGFP+ cells at distinct stages.** A** Heatmap showing 127 DECDGs between VSX2-eGFP+ cells at any two stages.** B** Heatmap showing the top 13 DECDGs between VSX2-eGFP+ and VSX2-eGFP- cells sorted at D28 and D55.** C** Bar chart showing expression of known RPC CD biomarkers CD24, FUT4, CD44, and KIT in VSX2-eGFP+ and VSX2-eGFP- cells sorted at D28 and D55. **D** Representative immunostaining images showed that KIT was expressed in Brn-3B+ retinal ganglion cells in addition to RPCs.** E** Bar chart showing the expression of TNFRSF1B in VSX2-eGFP+ and VSX2-eGFP- cells sorted at D28 and D55. **F** Representative immunostaining images showed the colocalization of TNFRSF1B with VSX2-eGFP+ cells sorted from early-stage (D31) ROs and seeded on coverslips for one day.** G** Representative immunostaining images showed that TNFRSF1B was expressed in the NRs from D35 to D55 and was progressively downregulated after D55. Scale bars: 100 μm (**D**,** G**), 50 μm (**F**)

To characterize the expression profile of CD biomarkers in human RPCs, we analyzed the dynamic expression patterns of 394 CD biomarkers listed by the Human Genome Organization Gene Nomenclature Committee (https://www.genenames.org) across various retinal developmental stages. A total of 6,256 DEGs derived from the comparison of VSX2-eGFP+ cells at any two distinct stages included 127 CD biomarker genes (DECDGs) (Fig. 6A). Thirty-nine DECDGs were enriched at D28, including biomarker genes associated with cell proliferation, differentiation, migration (*CD24*, *TNFRSF1B*, *TNFRSF10D*, *FGFR2*, and *FGFR3*), and adhesion (*NECTIN2*, *CD72*, *BCAM*, and *ICAM1*). Nine DECDGs were enriched at D55, encompassing biomarker genes related to tissue morphogenesis, axon guidance, cell motility, and migration (*IFITM1*, *HMMR*, *FZD10*, and *PLXNC1*). Twelve DECDGs, including biomarker genes related to neurogenesis, neurite outgrowth, and cell junctions (*NCAM1* and *NECTIN1*), were enriched at D118. Forty-one DECDGs, such as genes encoding the integrin alpha chain family of proteins (*ITGA1*, *ITGA2*, *ITGA3*, *ITGA5*, and *ITGB4*), were enriched at D170 (Fig. 6A).

Compared with those in VSX2-eGFP- cells, 13 CD biomarker genes, such as *TNFRSF1B*, *CD207*, *NT5E*, *KDR*, and *CD4*, were upregulated at D28 in VSX2-eGFP + cells, whereas 25 CD biomarker genes, such as *TLR9*, *VCAM1*, *CDH1*, *ACKR1*, and *IL1R1*, were downregulated. At D55, 49 CD biomarker genes, including *TNFRSF13C*, *TNFRSF1B*, *IFITM1*, *FZD10*, and *KDR*, were upregulated in VSX2-eGFP+ cells, whereas 17 CD biomarker genes, including *TLR9*, *ALK*, *L1CAM*, *THY1*, and *FCGR1A*, were downregulated (Fig. 6B).

Additionally, the expression patterns of CD biomarkers that have been reported to be expressed in mouse RPCs were tracked in human VSX2-eGFP+ cells at the very-early and early stages (D28 and D55). CD24, which labels RPCs from mice at E15 [13, 37], was highly expressed in VSX2-eGFP+ cells at D28 and then downregulated with differentiation (Fig. 6A). FUT4 (SSEA-1, CD15), a surface antigen used to define immature RPCs of the mouse retina [19, 20], was weakly expressed in VSX2-eGFP+ cells at all tested timepoints (FPKM < 2). CD44, a transiently expressed marker in mouse Kit+ RPCs exclusively differentiated into Müller glial cells [21], was increasingly expressed as the differentiation time increased (Fig. 6A). KIT (C-Kit, CD117), a widely used marker for identifying RPCs [10, 19], was enriched at D118 (Fig. 6A). Compared with those in VSX2-eGFP- cells, there were no significant differences in the CD24, FUT4, CD44, or KIT expression levels in VSX2-eGFP+ cells at the early stages, indicating that these are not specific markers for human RPCs (Fig. 6C). Immunostaining demonstrated that KIT was strongly expressed in Brn-3B+ retinal ganglion cells in addition to RPCs (Fig. 6D).

Furthermore, we attempted to identify biomarkers that have not been reported to be expressed in RPCs. We selected potential RPC markers based on three criteria: target-cell specificity (relative to VSX2-eGFP- populations), expression abundance (FPKM > 5), and biological relevance to RPC characterization. Through systematic screening, we identified TNFRSF1B, a member of the TNF-receptor superfamily that potentially mediates progenitor cell survival and neuroprotection [38–40], as a promising marker for sorting human RPCs. RNA-seq analysis revealed that TNFRSF1B was highly expressed (FPKM > 5) in VSX2-eGFP + cells at the very early/early stages (D28 and D55), but declined to near-undetectable levels (FPKM < 1) at later stages (intermediate/late) (D118 and D170) (Fig. 6A–B and E). VSX2-eGFP- populations at all tested time points exhibited undetectable TNFRSF1B expression (Fig. 6A and E). Next, we further validated the expression of the TNFRSF1B protein in early-stage ROs. Immunostaining confirmed that TNFRSF1B was expressed in VSX2-eGFP+ cells from early-stage ROs (Figs. 6F–G), and its expression level progressively decreased after D55 (Figs. 6G, S3A). Flow cytometry analysis of retinal cells isolated from early-stage ROs demonstrated TNFRSF1B positivity in 92 ± 5% of VSX2+ cells and 86 ± 7% of Ki67+ cells (Fig. S3B-D), supporting its utility as a candidate RPC biomarker.

## Discussion

Using hPSC-derived RPCs for cell replacement therapy is one of the most promising approaches for treating RDs. However, the cellular and molecular characteristics of human RPCs are not fully understood, especially the lack of surface markers for sorting RPCs, which has hindered their clinical application. In this study, we conducted a comprehensive analysis of the cellular and transcriptomic features of the human VSX2+ RPC population and reported significant heterogeneity at different developmental stages. Additionally, this study systematically analyzed the dynamic expression patterns of 394 CD biomarkers in VSX2-eGFP+ cells during human retinal development for the first time, and identified TNFRSF1B as a novel candidate surface marker for sorting RPCs. These data will provide valuable references for exploring genetic regulation of human retinal development and accelerating the development of cell therapy for RDs.

RDs have afflicted millions of people worldwide [1, 2]. Cell replacement constitutes one of the most promising therapeutic approaches for RDs, and hPSC-derived RPCs are among the preferred donor cells [5, 6, 10]. Nevertheless, the source of human RPCs is restricted due to the scarcity of human retinas. The rapid advancement of techniques for generating hPSC-derived ROs has offered an unlimited source of human RPCs for preclinical and clinical applications. In this study, we found that abundant numbers of proliferative human RPCs at very early and early developmental stages could be successfully obtained in the RO system, thereby establishing a robust foundation for subsequent research and clinical applications. Additionally, with the rapid progress of sequencing technology, the heterogeneity of RPCs has emerged as an international research focus [22–24]. Our study revealed that human VSX2+ RPCs displayed significant transcriptomic heterogeneity at different developmental stages, in accordance with the findings of other studies on mouse Vsx2+ RPCs [24]. These findings indicate that RPC subpopulations at appropriate developmental stages should be selected on the basis of the desired target cells during transplantation.

Despite the high-throughput utility of FACS for RPC sorting, clinically compatible surface markers for human RPCs remain scarce. Here, we present the dynamic expression profile of 394 CD biomarkers in human VSX2+ cells during retinal development, providing valuable references for the selective isolation of human RPCs. We found that some known RPC CD biomarkers, such as CD24, FUT4, CD44, and KIT [10, 19–21], were not specifically expressed in human VSX2+ RPCs at the very early and early stages, highlighting the critical need to validate marker expression using human-derived tissues and develop novel strategies (e.g., combining multiple markers or negative selection) to enhance RPC isolation purity. Future studies could adopt a multi-marker sorting strategy built upon previous research. For instance, THY1 can be used to exclude retinal ganglion cells, CD133 to eliminate photoreceptors, and SSEA4 to remove proliferative non-RPC populations [10, 14]. Additionally, our study may lay a foundation for identifying potential CD biomarkers for negative selection, such as L1CAM.

Additionally, our study endeavored to identify additional CD biomarkers to distinguish human RPCs. The protein encoded by the *TNFRSF1B* gene is a member of the TNF receptor superfamily and is involved in stimulating antioxidant pathways to protect neurons from cell death [38–40]. To our knowledge, this study is the first to report the expression of TNFRSF1B in human RPCs, which can provide more options for identifying and isolating human RPCs at very early and early stages. While TNFRSF1B’s neuroprotective roles in the central nervous system (CNS) have been partially documented [38–40], its function in retinal development remains unclear. In oligodendrocyte progenitor cells (OPCs), TNFRSF1B activation upregulates anti-apoptotic proteins (BCL-2, SOD2) through PI3K/Akt and NF-κB pathways, conferring resistance to oxidative stress [39]. Given the shared ontogeny of RPCs and OPCs as neural progenitors, TNFRSF1B likely employs similar mechanisms to protect RPCs from developmental stressors. Furthermore, in the CNS, TNFRSF1B activation shifts the balance from pro-inflammatory TNFR1 signaling toward regenerative responses [40], a mechanism that may extend to the retina. Future studies will include utilizing knockout models (e.g., *TNFRSF1B*-knockout hPSC-ROs) and pharmacological modulation (e.g., TNFRSF1B agonists/antagonists) to elucidate its functions in retinal development; such studies may also reveal novel therapeutic strategies for RDs characterized by progenitor cell dysfunction.

CD molecules not only serve as surface markers for cell identification and separation but also signal crucial events in retinal development. For example, biomarkers related to proliferation, differentiation, migration, and adhesion (CD24, TNFRSF1B, TNFRSF10D, FGFR2, FGFR3, NECTIN2, CD72, BCAM, and ICAM1) were enriched in VSX2-eGFP+ cells at D28 [37, 41–43], whereas biomarkers (IFITM1, HMMR, FZD10, and PLXNC1) related to tissue morphogenesis, axon guidance, cell movement, and migration were highly expressed in VSX2-eGFP+ cells at D55 [44–47], and biomarkers (NCAM1 and NECTIN1) related to neural development, neurite growth, and cell connections were highly expressed in VSX2-eGFP+ cells at D118 [48]. Therefore, the expression profile of the 394 reported CD biomarkers established here will also provide a valuable reference for research on retinal development and clinical disease diagnosis.

While our study provides valuable insights, there exist several limitations. For instance, we observed a minor imperfect overlap between the expression patterns of eGFP and VSX2 in early-stage ROs, where a trace of transient eGFP signals was detected in the innermost (retinal ganglion cell-like) layer of the NRs, thus affecting the purity of sorted eGFP+ populations to a certain degree. This mismatch was absent in the very-early stages, diminished progressively with cell maturation, and became minimal in late stages. Therefore, we speculate that the transient VSX2-eGFP signals may arise from the transition of VSX2-eGFP+ RPCs to their daughter cells because fluorescent proteins are more stable than endogenous proteins [49]. A similar phenomenon has also been observed in previous studies, including research related to ROs [50, 51]. However, the exact reason remains unclear. In future studies, single-cell RNA-seq or multiple CD markers could be used to resolve intrapopulation heterogeneity among eGFP+ cells.

## Conclusions

In conclusion, the rapid progress in hiPSC/ESC-derived RO techniques and fluorescent reporter systems provides an accessible platform for directly investigating the cellular and molecular characteristics of human RPCs. Our study provides valuable insights into the molecular and cellular characteristics of human RPCs, especially their expression profiles of CD biomarkers, laying a foundation for research on retinal development and the clinical translation of hiPSC-derived RPCs. Further studies will build upon these discoveries to sort RPCs for transplantation research.

## Supplementary Information

Below is the link to the electronic supplementary material.

Supplementary Material 1

## Data Availability

All the data generated or analyzed during this study are included in this published article and its supplementary information files. The accession number for the RNA-seq data reported in this paper is GEO: GSE185475.

## Abbreviations

RD: Retinal degenerative disease
RPC: Retinal progenitor cell
hPSC: Human pluripotent stem cell
hESC: Human embryonic stem cell
hiPSC: Human induced pluripotent stem cell
RO: Retinal organoid
VSX2: Visual system homeobox 2
eGFP: Enhanced green fluorescent protein
D: Differentiation day
+: Positive
-: Negative
RT‒qPCR: Real-time quantitative polymerase chain reaction
FACS: Fluorescence-activated cell sorting
RNA-seq: RNA sequencing
FPKM: Fragment per kilobase of transcript per million mapped reads
FDR: False discovery rate
DEGs: Differentially expressed genes
GSEA: Gene set enrichment analysis
NES: Normalized enrichment score
NOM: Nominal
EFs: Eye field-like structures
OCs: Optic cup-like structures
NR: Neural retina
RPE: Retinal pigment epithelium
SEGs: Specifically expressed genes
DECDGs: Differentially expressed CD biomarker genes
