# Supplementary material for: Dynamic molecular and cellular characteristics of VSX2-positive retinal progenitor cells in human retinal organoids
Source: Stem Cell Res Ther. 2025 Oct 28;16:589. doi: 10.1186/s13287-025-04700-z (PMC12570740; doi:10.1186/s13287-025-04700-z)
Supplement: Supplementary file 1 — Supplementary Material 1 [file 13287_2025_4700_MOESM1_ESM.docx]

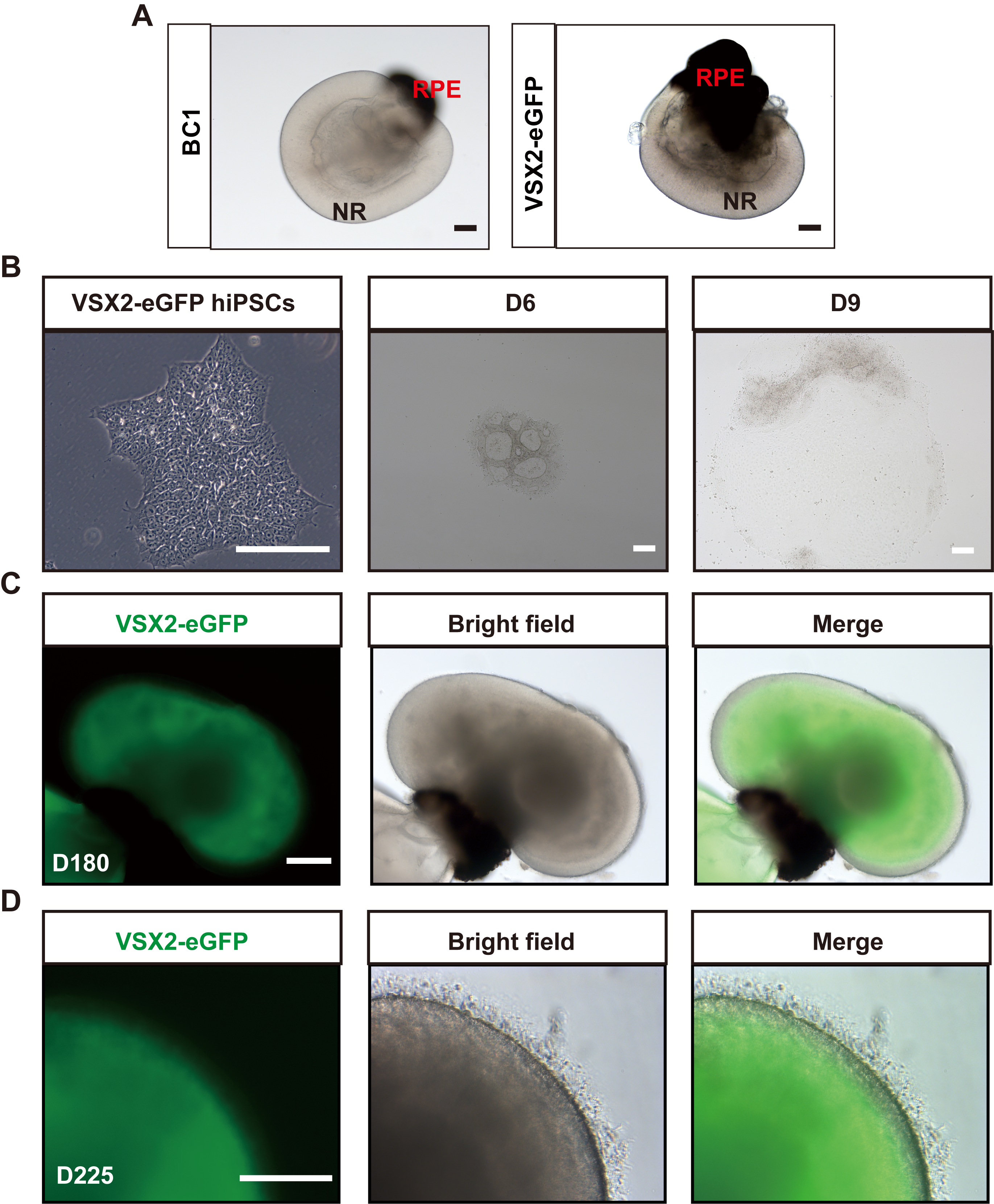


**Fig. S1** **Spatiotemporal expression of the VSX2-eGFP reporter in the RO system.** (**A**) Bright-field images of the reporter and parental hiPSC-derived ROs. (**B-D**) Fluorescent and bright-field images showing the spatiotemporal expression patterns of VSX2-eGFP reporter in the RO system from D0 to D225. Scale bars: 100 μm (A); 200 μm (B-D).


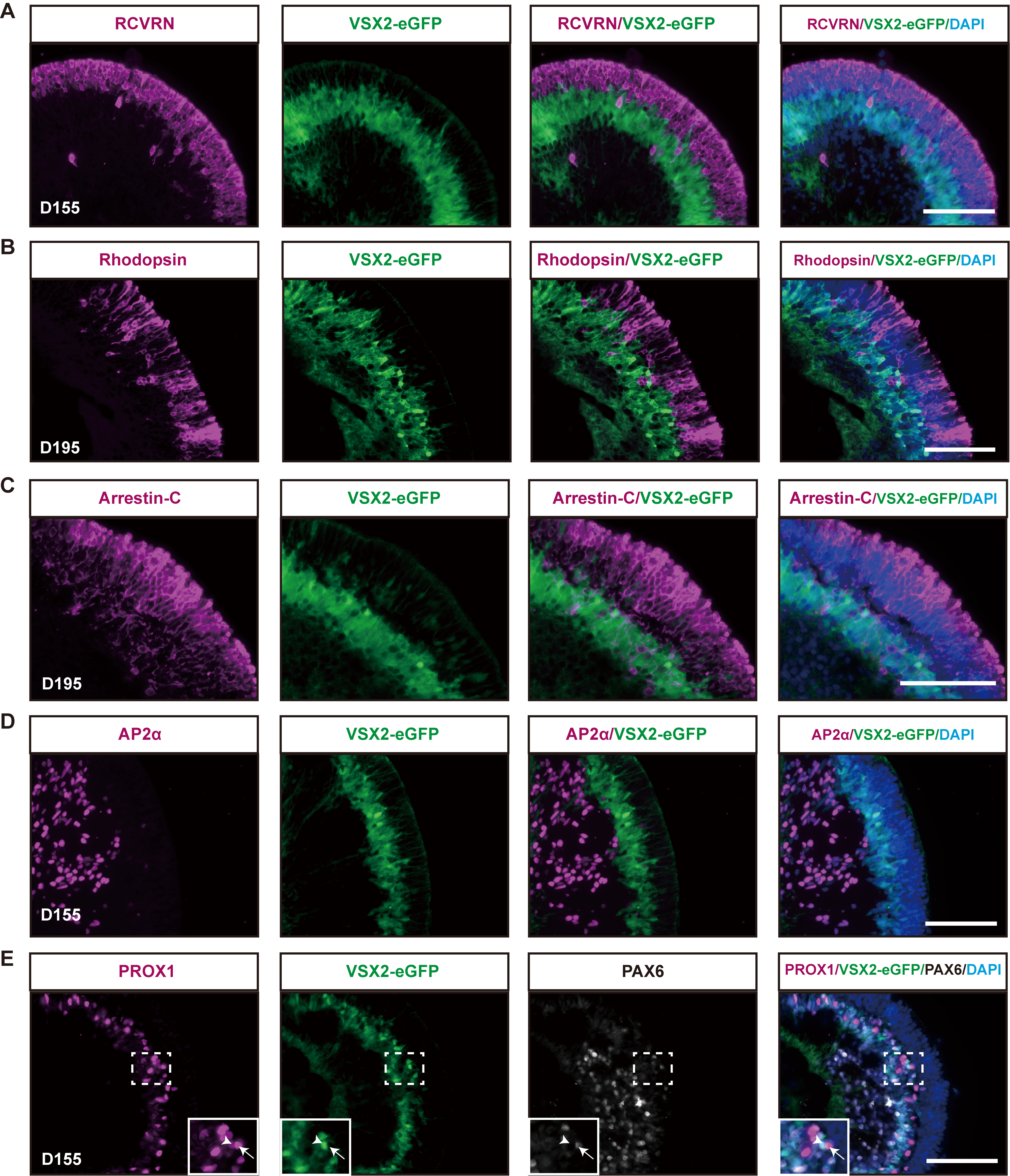


**Fig. S2 Cellular characteristics of VSX2-eGFP+ cells at late stages.** **(A-E)** Immunostaining showing the expression of photoreceptor markers RCVRN, Rhodopsin (rod), Arrestin-C (cone), amacrine cell marker AP2α and horizontal cell markers PROX1 and PAX6 in VSX2-eGFP hiPSC-derived ROs. Arrows highlight VSX2-eGFP- horizontal cells (PROX1+ and PAX6+), while arrowheads indicate VSX2-eGFP+ bipolar cells (PROX1+ and PAX6-). Scale bars: 100 μm.


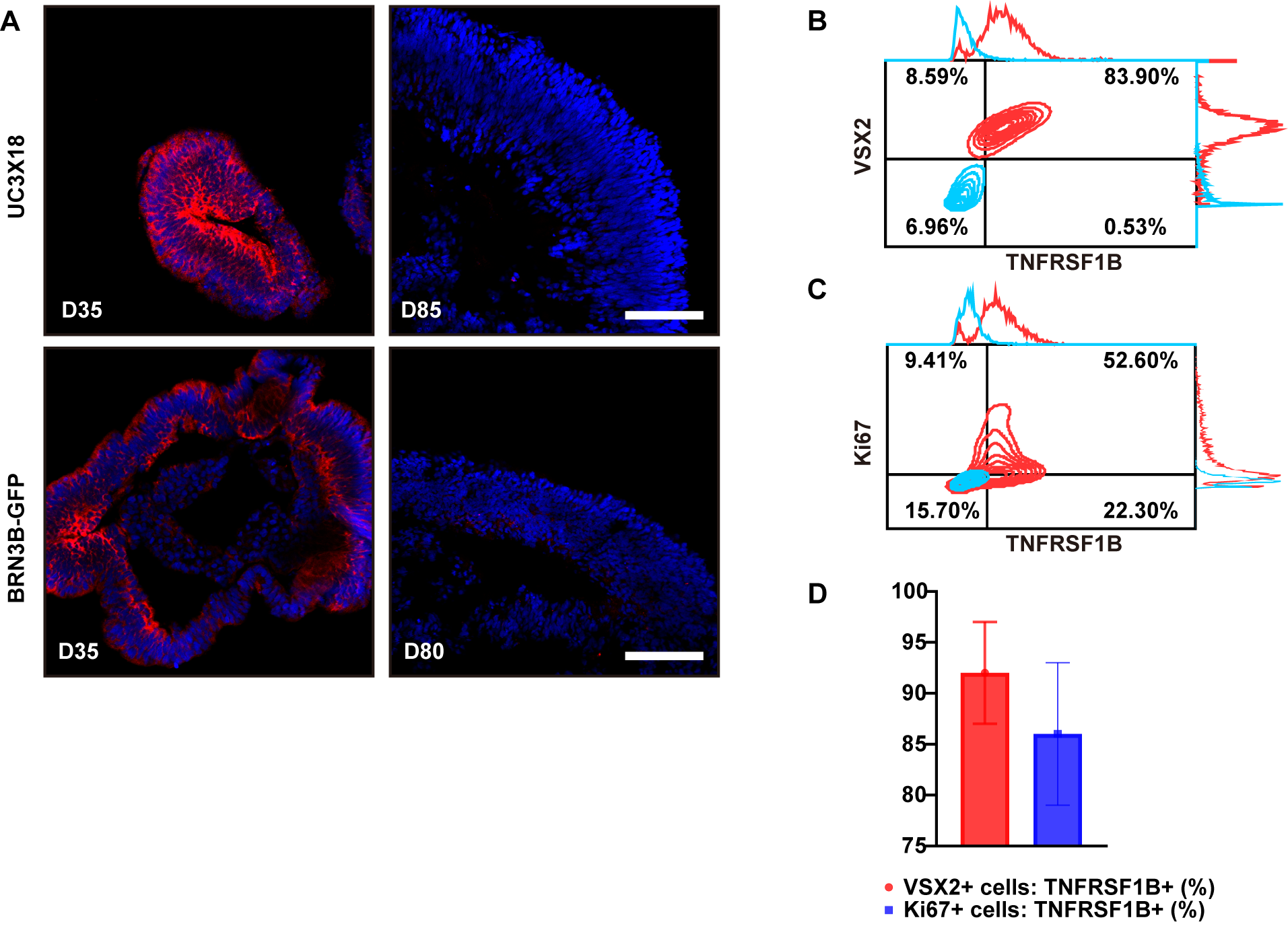


**Fig. S3** **The Expression of TNFRSF1B in ROs at early stages.** **(A)** Representative immunostaining images revealed consistent TNFRSF1B expression in the NR at early stages across ROs derived from different hiPSC lines. (B-D) Flow cytometry analysis of retinal cells isolated from ROs at D55 demonstrated TNFRSF1B positivity in 92 ± 5% of VSX2+ cells and 86 ± 7% of Ki67+ cells, n = 4. Scale bars: 100 μm (A).

­

**Table S1. A list of antibodies used for immunostaining.**

| **Antigen** | **Supplier** | **Catalog Number** | **Host** | **Dilution** |
| --- | --- | --- | --- | --- |
| AP2a | DSHB | 3B5-S | Mouse | 1:35 |
| BRN-3B | Santa Cruz | sc-6026 | Goat | 1:100 |
| VSX2 | Millipore | ab9016 | Sheep | 1:200 |
| OTX2 | Abcam | ab21990 | Rabbit | 1:500 |
| Ki67 | Abcam | ab15580 | Rabbit | 1:200 |
| PAX6 | DSHB | AB528427 | Mouse | 1:50 |
| PKCа | Abcam | ab32376 | Rabbit | 1:2000 |
| PROX 1 | Millipore | AB5475 | Rabbit | 1:2000 |
| RCVRN | Millipore | ab5585 | Rabbit | 1:500 |
| RAX | Santa Cruz | sc-271889 | Mouse | 1:200 |
| SOX1 | Boster | BM4661 | Rabbit | 1:50 |
| SOX9 | ABclonal | A19710 | Rabbit | 1:250 |
| Arrestin-C | Novus | NBP1-37003 | Goat | 1:200 |
| Rhodopsin | Abcam | ab3267 | Mouse | 1:200 |
| KIT | Boster | PB9258 | Rabbit | 1:500 |
| TNFRSF1B | Thermo Scientific | MA5-32618 | Rabbit | 1:200 |
| MCM2 | Abcam | ab4461 | Rabbit | 1:1000 |

**Table S2. Primers Used for RT- qPCR.**

| **Gene amplified** | **Forward** | **Reverse** | **Size (bp)** |
| --- | --- | --- | --- |
| *GAPDH* | *TGCACCACCAACTGCTTAGC* | *GGCATGGACTGTGGTCATGAG* | 87 |
| *NANOG* | *AAGGTCCCGGTCAAGAAACAG* | *CTTCTGCGTCACACCATTGC* | 237 |
| *GDF3* | *AAATGTTTGTGTTGCGGTCA* | *TCTGGCACAGGTGTCTTCAG* | 179 |
| *DNMT3B* | *ATAAGTCGAAGGTGCGTCGT* | *GGCAACATCTGAAGCCATTT* | 203 |
| *SOX2* | *GACAGTTACGCGCACATGAA* | *TAGGTCTGCGAGCTGGTCAT* | 176 |
| *OCT4* | *AACCCACACTGCAGCAGATCA* | *TCTCGTTGTGCATAGTCGCT* | 127 |
